# Diagnostic dilemma of IgG4-related primary localized cervical lymphadenopathy associated with aberrant IL-6 expression level

**DOI:** 10.1186/s13000-016-0493-3

**Published:** 2016-05-03

**Authors:** Moriyoshi Nakamura, Osamu Iwamoto, Takahiro Chino, Keita Todoroki, Jingo Kusukawa

**Affiliations:** Dental and Oral Medical Center, Kurume University School of Medicine, 67 Asahi-machi, Kurume, 830-0011 Japan; Department of Biomedical Sciences, University of the Pacific, Arthur A. Dugoni School of Dentistry, 155 fifth St, San Francisco, CA 94103 USA

**Keywords:** IgG4, IgG4-related disease, Lymphadenopathy, IL-6, Castleman’s disease

## Abstract

**Background:**

Immunoglobulin G4-related disease (IgG4-RD) is a recently recognized inflammatory condition with single- or multi-organ involvement. The disease is characterized by tumefactive lesions with dense IgG4 plasmacytic infiltration (an elevated IgG4^+^/IgG^+^ cell ratio of > 40 %), storiform fibrosis, and obliterative phlebitis, with or without elevated serum IgG4 levels. The diagnostic criteria for IgG4-RD, proposed in 2011, were quite comprehensive and practical; however, it is important to remember that other diseases, such as hyper-interleukin (IL)-6 syndromes, may have common histopathological findings. Therefore, the histopathology of suspected IgG4-RD is occasionally not diagnostic. Here, we report a case of IgG4-related primary localized cervical lymphadenopathy without any other organ involvement. To our knowledge, there have been no previous reports of this. Additionally, the disease was associated with a 20-fold increase in IL-6 levels compared to that of the normal range.

**Case presentation:**

We report the case of a 52-year-old Japanese man who presented with a painless, somewhat diffuse swelling in the left submandibular region. Although the case fulfilled diagnostic criteria for IgG4-RD, the diagnosis was not straightforward due to abnormally high levels of serum IL-6. After systematic evaluation of the patient, a final diagnosis of IgG4-RD was established. Since then, a specialist in connective tissue disorders has evaluated the patient on a regular basis. Two years after his initial visit, no disease progress or systemic involvement has been noted.

**Conclusion:**

We present a case of an IgG4-related primary localized cervical lymphadenopathy mimicking hyper-IL-6 syndrome. This case can serve as an excellent reminder that the definitive diagnosis of IgG4-RD should be established using a systematic approach, in particular when it appears as an atypical manifestation.

## Background

Swellings of the cervical area are often associated with congenital or acquired conditions including cystic, inflammatory, infectious, and neoplastic diseases. Thus, the differential diagnosis of diseases involving cervical swelling is quite extensive. Immunoglobulin G4-related disease (IgG4-RD) is a recently recognized inflammatory condition that has single- or multi-organ involvement. The head and neck region is the second most common site for the development of IgG4-RD. The disease is characterized by tumefactive lesions with dense IgG4 plasmacytic infiltration (an elevated IgG4^+^/IgG^+^ cell ratio of > 40 %, and > 10 IgG4^+^ cells per high power field), storiform fibrosis, and obliterative phlebitis with or without elevated serum IgG4 levels [1–3]. The presence of these three histopathological findings, as well as the increased number and ratio of IgG4^+^ plasma cells, is highly suggestive of a diagnosis of IgG4-RD [4]. Although lymphadenopathy is frequently associated with IgG4-RD, it usually lacks storiform fibrosis, and its histopathological findings are further divided into five types. These include multicentric Castleman’s disease-like (type I), reactive follicular hyperplasia-like (type II), interfollicular expansion and immunoblastosis (type III), progressively transformed germinal center (PTGC) type (type IV), and inflammatory pseudotumor like (type V) IgG4-related lymphadenopathy [5].

Increased numbers of IgG4^+^ plasma cells might be associated with non-IgG4-RD, such as low-grade B-cell lymphomas and hyper-interleukin (IL)-6 syndromes, such as Castleman’s disease [6] and rheumatoid arthritis [7], all of which can result in cervical lymphadenopathy. Since histopathological findings of such diseases are occasionally similar to that of IgG4-RD, Sato and Yoshino [5] proposed that the combination of histological examination and laboratory analyses are essential for the definitive diagnosis of the disease.

Here, we report a case of IgG4-related primary localized cervical lymphadenopathy without any other organ involvement. To our knowledge, there have been no previous reports of this. In addition, the disease was associated with 20-fold higher IL-6 levels than those of the normal range.

## Case presentation

A 52-year-old Japanese male with no significant past medical history visited our clinic in 2013 for evaluation of a swelling in the left submandibular region, which had increased in size over four years. The patient had not experienced any symptomatic manifestations.

**Fig. 1:**
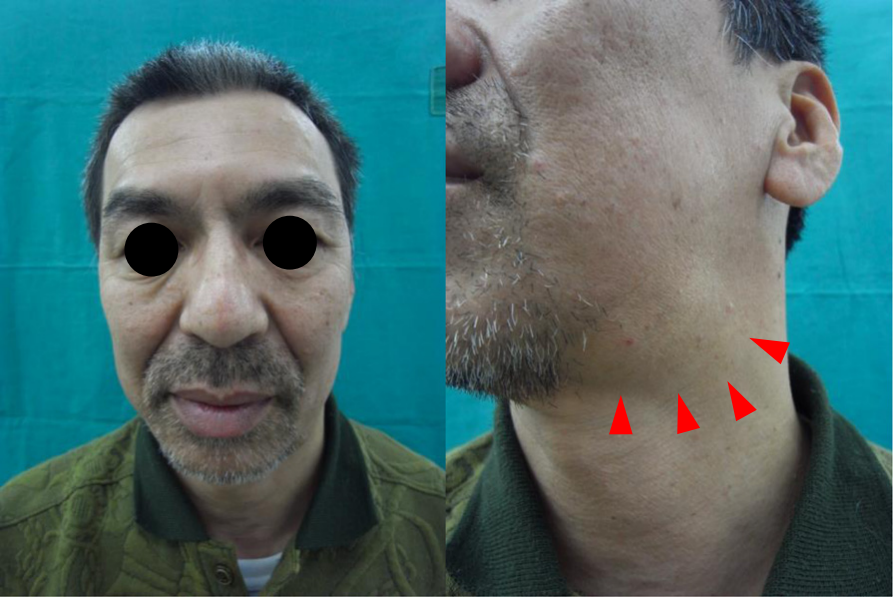
The first extraoral photograph. Extraoral examination revealed a 40 × 20 mm mass (arrowhead) in the left submandibular region that was elastically hard, movable, painless, and covered with normal skin

**Fig. 2:**
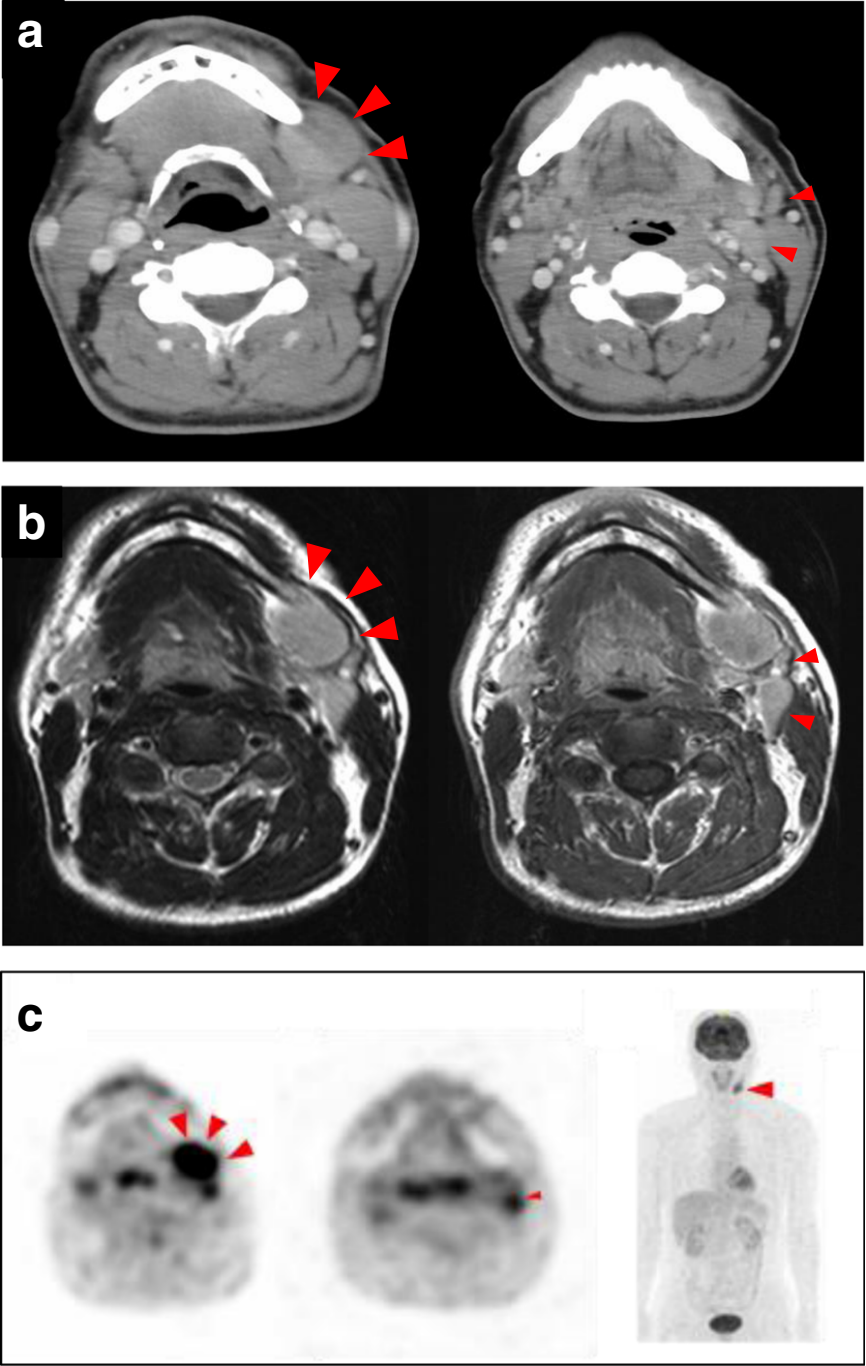
CT scan (**a**) and Contrast-enhanced T1-weighted MRI (**b**) revealed an oval swelling (*Large arrowhead*) in the left submandibular region associated with enlarged submental lymph nodes (*Small arrowhead*) and the superior internal jugular area. A PET (**b**) revealed abnormal accumulation of FDG in the left submandibular (*Large arrowhead*) and left upper internal jugular regions (*Small arrowhead*). No clear abnormal accumulation was noted in any area other than the neck region

**Fig. 3:**
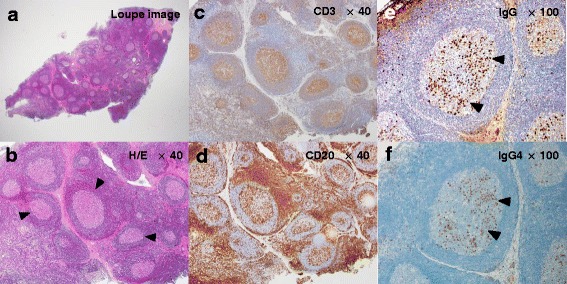
**a**, **b** Histopathological specimens revealed lymphatic follicles (*arrowhead*) with an enlarged and hyperplastic germinal center. **c**, **d** The specimens were immunoreactive against both CD3 and CD20. **e**, **f** Immunohistochemical staining of IgG and IgG4 revealed a ratio of IgG4/IgG-positive plasma cells (*arrowhead*) of approximately 50 % and large quantities of IgG4 were noted in the germinal center

**Fig. 4:**
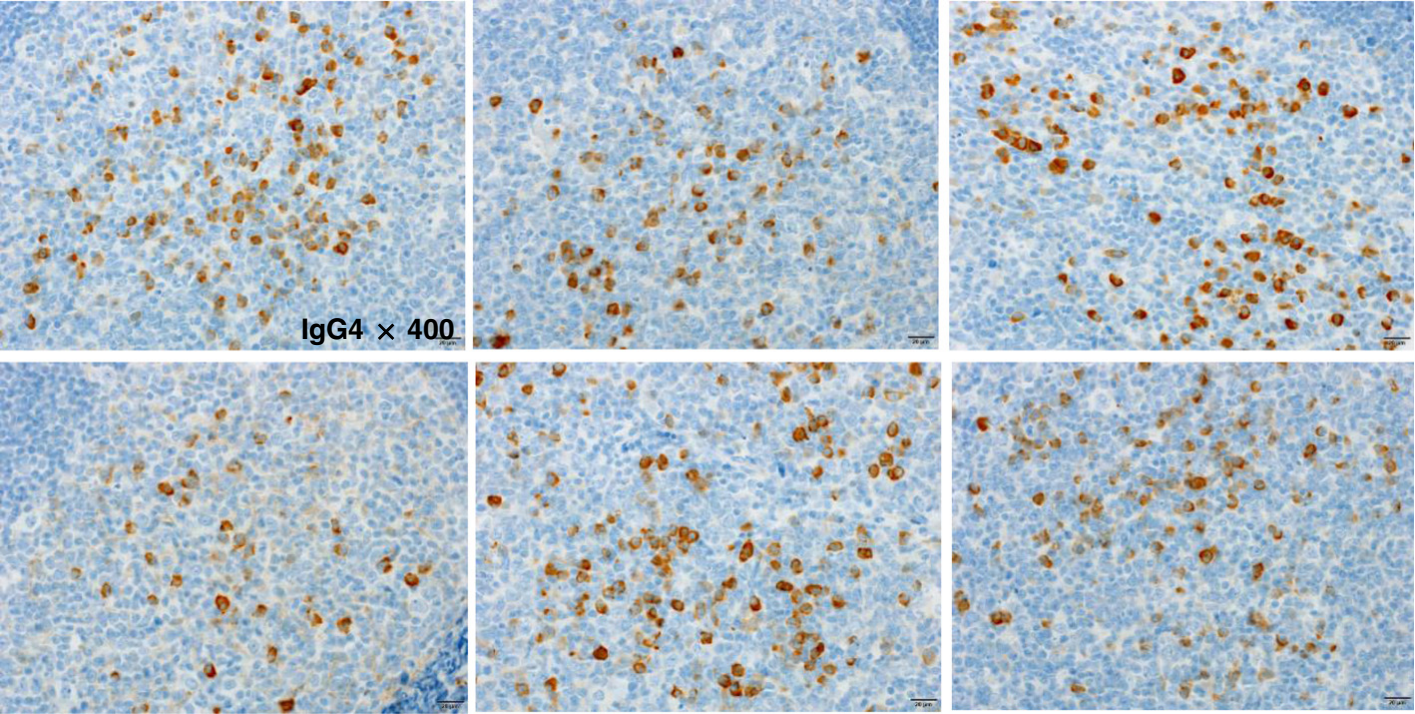
The mean number of IgG4+ plasma cells per high power field was 94 (original magnification 400 ×)

Extraoral examination revealed a 40 × 20 mm mass in his left submandibular region that was elastically hard, movable, painless, and covered with normal skin (Fig. 1). Intraoral examination revealed an appropriate salivary flow, and therefore, the patient was not xerostomic. A computed tomography (CT) scan revealed a 35 × 23 mm oval swelling in the left submandibular region associated with enlarged submental lymph nodes and superior internal jugular area, in which contrast medium was taken up homogenously (Fig. 2a). Contrast-enhanced T1**-**weighted magnetic resonance imaging (MRI) demonstrated enlarged submandibular and submental lymph nodes and ipsilateral upper internal jugular vein (Fig. 2b). Additionally, in T2 weighted MRI, they structures were hypointense. Positron emission tomography (PET) revealed abnormal accumulation of fluorodeoxyglucose (FDG) in the left submandibular and left upper internal jugular regions. The FDG-standardized uptake value (SUV) max values were 5.09–8.24 for the left submandibular and 2.82–3.19 for the left upper internal jugular area. No clear abnormal accumulation was noted in any area other than the neck region (Fig. 2c), and laboratory tests revealed no inflammation. Furthermore, IL-2R and LDH values were normal (Table 1). Based on clinical, imaging, and laboratory findings, the patient was diagnosed with malignant lymphoma. However, the fine needle aspiration cytology (FNAC) of the swollen lymph node failed to show signs of malignancy. Subsequent biopsy revealed lymphatic follicles with an enlarged and hyperplastic germinal center. Histiocytes, lymphocytes, and plasma cells were scattered between the follicles. Histopathological specimens also revealed a normal shape of the lymphocytic nucleus as well a normal nucleus-cytoplasmic ratio (Figs. 3a and b). The specimens were immunoreactive against both CD3 and CD20 antibodies (Figs. 3c and d), and were negative for the neoplastic lesion. Although reactive follicular hyperplasia is associated with pathological conditions, such as rheumatic disorders, systemic lupus erythematosus and bacterial lymphadenitis were included as differential diagnoses, as rheumatoid factor (RF), anti-DNA antibody, and C-reactive protein (CRP) values were all within the normal range (Table 2). However, increased serum IgG4 (Table 2) indicated the possibility of IgG4-related lymphadenopathy. Immunohistochemical staining against IgG and IgG4 revealed a ratio of IgG4/IgG-positive plasma cells of approximately 50 % and > 10 IgG4 cells per high power field, noted in the germinal center (Figs. 3e, f and 4). These findings fulfilled the diagnostic criteria issued in 2011 for IgG4-related disease [7], although the serum level of IL-6 was 20-fold higher than the normal value.

**Table 1:** Laboratory tests

General	Biochemical	Special method inspection
WBC 5900/μL	AST 18 U/L	IL2R 382 U/mL
Neut 50.2 %	ALT 25 U/L	CEA 2.1 ng/mL
Eos 2.2 %	LD 15 U/L 0	SCC 1.0 ng/mL
Bas 0.5 %	ALP195 U/L	
Mon 7.3 %	r-GTP 42 U/L	
Lym 39.8 %	TP 7.31 g/dL	
RBC 521 × 10^4^ /μL	Alb 4.42 g/dL	
Hb 16.3 g/dL	BUN 18.2 mg/dL	
Hct 47.2 %	Cre 0.76 mg/dL	
PLT 27 × 10^4^ /μL

**Table 2:** Laboratory tests

IgA221 mg/dL	RF <1 IU/mL
IgM 157 mg/dL	Anti SS-A/Ro antibody(-)
IgG 1136 mg/dL	Anti SS-B/La antibody (−)
IgG4 153 mg/dL ↑	Anti DNA antibody (−)
CRP 0.11 mg/dL	Anti RNP antibody (−)
IL −6 83.5 pg/mL ↑	MPO. ANCA <1.0
	PR3-ANCA <1.0

Based on the final diagnosis of IgG4-related cervical lymphadenopathy (type II reactive follicular hyperplasia type), the patient was referred to the Department of Internal Medicine and Connective Tissue Disorders, Kurume University School of Medicine, two months after his initial visit. The patient has been under regular follow-up, and no disease progress or systemic involvement has been noted.

## Discussion

IgG4-RD is a recently established immune-mediated, inflammatory condition that affects virtually every organ system. Lymphadenopathy, often associated with extranodal lesions, is frequently observed in patients with IgG4-RD. Lymph node involvement often occurs after or at the same time as the development of the extranodal lesion. The initial unifocal lymphadenopathy may progress to a multifocal lesion and/or develop to have extranodal involvement [8]. The present case is somewhat atypical as an IgG4-RD in two aspects: 1) unifocal cervical lymphadenopathy, and 2) association of high serum IL-6.

In the report by Sato et al. [5], IgG4-related lymphadenopathy histologically was divided into I-V types. In many cases, in the type similar to Castleman’s disease and ALPIBP type, lymph node swelling occurs throughout the body. However, with the reactive follicular hyperplasia type (the type similar to inflammatory pseudotumors and the PTGC-like type), most cases appear to have localized swelling of the lymph nodes. The IgG4-positive cell permeation pattern is intra-follicular in cases similar to Castleman’s disease, the reactive follicular hyperplasia type, the ALPIBP type, and the type similar to inflammatory pseudotumors, while with the PTGC-like type, there is a greater incidence of germ center permeation. In this case, the patient experienced localized swelling of the lymph nodes, and histopathological testing revealing that the IgG4-positive plasma cells had permeated the germ center; therefore, it was initially believed to be the PTGC-like type. Despite this, the fact that the follicle structure of the germ center had been preserved indicated that there was no progressive dysplasia, although hyperplastic deformation of the large and small lymphatic follicles was noted. It was eventually determined that the condition was the reactive follicular hyperplasia type.

The head and neck region, including salivary, lacrimal, thyroid, and pituitary glands is the second most common site affected by IgG4-RD [9]. IgG4-RD in the head and neck region can be an extrapancreatic manifestation associated with autoimmune pancreatitis or can present as a solitary lesion. Mikulicz disease and chronic sclerosing sialadenitis, often associated with lymphadenopathy, are the most common IgG4-RDs in the head and neck area. However, the disease presented here was still localized to the cervical area with and no extranodal involvement has been noted since the patient was initially diagnosed with IgG4-RD.

The diagnostic dilemma in the present case was an extremely high level of serum IL-6 since hyper-IL-6 syndromes occasionally fulfill the histopathological diagnostic criteria for IgG4-RD and have elevated serum IgG4 levels [3, 4]. A few cases of IgG4-related systemic lymphadenopathy with elevated (~9-fold) IL-6 levels have been reported [2, 10]. However, the present case was associated with a serum IL-6 level 20-fold higher than the normal range. This is the first case of IgG4-RD cervical lymphadenopathy associated with such a high level of IL-6. IL-6 was originally cloned as T cell-derived B cell activating molecule in 1986 [11]. Currently, it is known as a pleiotropic cytokine playing a role in the regulation of immune system, osseous tissues, and inflammation. It also has a role as a growth factor in malignancies, such as multiple myeloma (MM) [12]. The variation of IL-6 levels in individuals and/or the circadian rhythm of serum IL-6 may account for the elevated IL-6 level in the present case. It has been suggested that IL-6 values might vary widely among healthy individuals [10]. In the pathological situation, patients with MM reveal elevated, normal, or undetectable serum levels of IL-6. Additionally, circadian variation of serum IL-6 has been reported in several diseases [12–14].

**Fig. 5:**
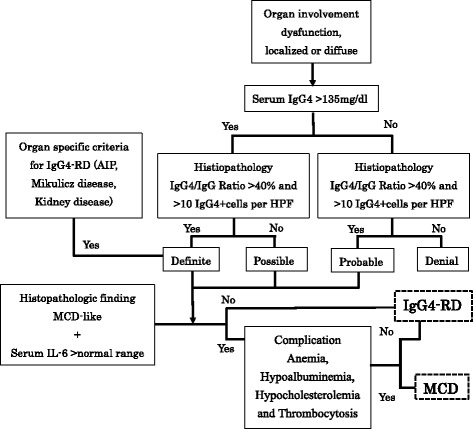
Comprehensive diagnostic criteria for IgG4- related disease (modified after Umehara et al. [2])

**Fig. 6:**
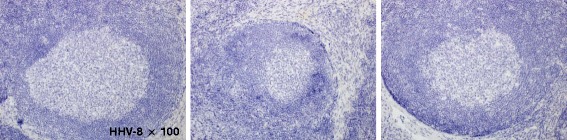
Histopathological specimens were negative for HHV-8 immunohistochemical staining (original magnification 100 ×)

Both IgG4-RD and hyper-IL-6 syndrome share similar clinical and pathological manifestations and, therefore, it is quite difficult to differentiate them. As suggested by Sato et al. [5], diagnosis of IgG4-RD must be established based on histopathological appearance as well as increased serum IgG4. However, occasional increases in serum IL-6 and IgG4 in IgG4-RD and hyper-IL-6 syndrome, respectively, make the differentiation between IgG4-RD and hyper-IL-6 syndrome more difficult. Hyper-IL-6 syndromes are rheumatoid arthritis, Sjögren’s syndrome, systemic lupus erythematosus, mixed connective tissue disease, human immunodeficiency virus (HIV) infection, human herpes virus 8 (HHV8) infection, Kaposi’s sarcoma, plasma cell dyscrasia, Hodgkin’s lymphoma, and malignant lymphoma. In particular, the histological findings of multicentric Castleman’s disease (MCD) and IgG4-related lymphadenopathy are often similar. MCD is a distinct type of lymphoproliferative disorder associated with inflammatory symptoms and IL-6 dysregulation. In the context of HIV infection, MCD is associated with HHV8 infection [15, 16]. Anemia, hypoalbuminemia, hypocholesterolemia, and thrombocytosis are peculiar to hyper-IL-6 syndrome, and were not found in the present case (Fig. 5) [6]. Furthermore, we could not detect HHV8 in the lymph nodes (Fig. 6), ruling out HHV8-associated MCD.

### Consent

Written informed consent was obtained from the patient for publication of this Case Report and any accompanying images. A copy of the written consent is available for review by the Editor-in-Chief of this journal.

## Conclusions

We present a case of an IgG4-related primary localized cervical lymphadenopathy mimicking hyper-IL-6 syndrome. This case can serve as an excellent reminder that the definitive diagnosis of IgG4-RD should be established using a systematic approach, in particular when it appears as an atypical manifestation.

## Abbreviations

CRP: C-reactive protein
CT: computed tomography
FDG: fluorodeoxyglucose
FNAC: fine needle aspiration cytology
HHV8: human herpes virus 8
HIV: human immunodeficiency virus
IgG4-RD: Immunoglobulin G4-related disease
MCD: multicentric Castleman’s disease
MM: multiple myeloma
MRI: magnetic resonance imaging
PET: positron emission tomography
PTGC: progressively transformed germinal center
RF: rheumatoid factor
SUV: standardized uptake value
